# Quantitative conversations: the importance of developing rapport in standardised interviewing

**DOI:** 10.1007/s11135-014-0144-2

**Published:** 2014-12-25

**Authors:** Karen Bell, Eldin Fahmy, David Gordon

**Affiliations:** School for Policy Studies, University of Bristol, 8, Priory Road, Bristol, BS8 1TZ UK

**Keywords:** Surveys, Paradata, Behaviour coding, Conversational interviewing, Standardised interviewing, Rapport

## Abstract

When developing household surveys, much emphasis is understandably placed on developing survey instruments that can elicit accurate and comparable responses. In order to ensure that carefully crafted questions are not undermined by ‘interviewer effects’, standardised interviewing tends to be utilised in preference to conversational techniques. However, by drawing on a behaviour coding analysis of survey paradata arising from the 2012 UK Poverty and Social Exclusion Survey we show that in practice standardised survey interviewing often involves extensive unscripted conversation between the interviewer and the respondent. Whilst these interactions can enhance response accuracy, cooperation and ethicality, unscripted conversations can also be problematic in terms of survey reliability and the ethical conduct of survey interviews, as well as raising more basic epistemological questions concerning the degree of standardisation typically assumed within survey research. We conclude that better training in conversational techniques is necessary, even when applying standardised interviewing methodologies. We also draw out some theoretical implications regarding the usefulness of the qualitative–quantitative dichotomy.

## Introduction

To increase survey accuracy, research designers now place considerable emphasis on developing better survey instruments. However, although the quality of the question items is a key determinant of overall survey quality and ethicality, interviewer behaviour is a key factor often overlooked in practical assessments of survey data quality. Nevertheless, numerous studies have shown interviewers’ impact on data quality and survey cooperation as a function of: their personal characteristics and experience; their psychological dispositions, such as their expectations, perceptions and motives; and their behaviour, including their method of communication (e.g. Olson and Peytchev 2007). In this paper, we focus on the last of these ‘interviewer effects’—interviewer behaviour. There is substantial evidence to indicate that the behaviour of survey interviewers influences not only whether answers will be accurate and honest (e.g. Schaeffer et al. 2010), but also whether respondents will agree to answer survey questions at all (e.g. Lipps and Pollien 2011), and whether or not there are negative impacts from respondents’ involvement in the research (Lewis and Graham 2007).

A growing realisation of the importance of interviewer behaviour on survey response has reinforced a preference for standardised interviewing techniques over conversational interviewing methods, so that the researcher can maintain control over the interviewer–respondent interaction. Where conversational interviewing is still encouraged, there remains an ongoing and unresolved debate regarding the most appropriate interviewer behaviour for enhancing survey accuracy, cooperation and ethicality. Hence, we discuss both the extent and content of interviewer effects and link this to key debates regarding standardised versus flexible interviewing and appropriate conversational techniques, such as building rapport. The extent and nature of interviewers’ deviations from the standardised scripts are then explored and illustrated by drawing upon interview paradata collected as part of the *2012 UK Poverty and Social Exclusion Survey* (http://www.poverty.ac.uk/). This analysis reveals that deviations from the standardised approach are widespread, and that some unscripted conversations may undermine the accuracy or comprehensiveness of responses or even be potentially harmful to respondents. We then go on to offer some concluding observations on the implications of our findings for research practice, in terms of further survey interviewer training, and for wider methodological debates concerning the dichotomisation of quantitative and qualitative techniques.

## Standardised versus flexible interviewing

With standardised interviewing, interviewers are required to read questions exactly as written. Probing is expected to be nondirective and feedback to be as neutral as possible. For example, when respondents seek clarification they will be offered standardised responses such as ‘please answer according to your understanding of this term’. Standardised interviewing is considered to reduce interviewer related error through ensuring that all respondents are asked identically worded questions without unscripted commentary that could bias answers (Fowler and Mangione 1990). From a more practical perspective, it is also argued that standardised interviewing reduces costs by minimizing the interview length, interviewer training time, and coding time (Fowler and Mangione 1990). For these reasons, most survey organisations maintain a strong preference for standardisation and data is collected using scripted interviews.

However, Fowler and Mangione (1990) have suggested that interview standardisation could also constrain the development of rapport between interviewer and respondent. They consider that it is difficult to administer household surveys in a completely standardised way since some (unscripted) conversational interaction between interviewer and respondent is essential. Furthermore, Conrad and Schober (1997) assert that it is not possible to guarantee a uniform understanding of survey questions using a strictly standardised approach. They argue that less structured forms of interviewing, such as conversational interviewing, often increase data accuracy because they facilitate standardisation of the *meaning* of questions, which is not possible when interviewers adhere strictly to scripted questions. In conversational interviewing, the interviewer says what is necessary to help the respondents to correctly interpret the question. Conrad and Schober (1997) found that conversational interviewing improves the accuracy of responses when interviewees’ circumstances are atypical:

Respondents answer accurately about typical situations, whether or not interviewers are licensed to clarify the concepts in the questions. However, when respondents answer about atypical situations, their accuracy depends on whether or not they are able to get clarification from the interviewers. When they cannot get this help, they are very inaccurate; when they can obtain it their accuracy improves. The more flexibly the interviewers can provide help, the more it improves response accuracy (Conrad and Schober 1997, no page number).

Similarly, Dykema et al. (1997) found that interviewers who change question wording obtain more accurate answers when the questions are more complex than those who do not and suggest that interviewers predict which problems may be problematic and alter these questions in advance so as to ensure a correct interpretation. Respondents may not seek clarification when required, so it may be helpful if interviewers identify verbal or non-verbal signs that respondents need clarification, even where this is not explicitly requested. Furthermore, flexibility when interviewing appears productive in gaining co-operation. Interviewers that are good at soliciting cooperation from respondents are usually also skilled at tailoring their approach to the situation (Morton-Williams 1993).

Whether flexible or standardised interviewing is the aim, it seems that in practice survey interviewers frequently deviate from the scripted wording (Cannell et al. 1981). Some of these deviations appear to be unhelpful, including failing to ask some scheduled questions, giving feedback which could influence future responses, and using directive probes (ibid). For example, interviewers sometimes give positive feedback even when respondents do not give adequate answers so as to maintain a harmonious interaction (ibid). Some studies show that the more experience interviewers gain, the more likely they are to deliver survey questionnaires in a less standardised way (e.g. Pickery and Loosveldt 2001; Olson and Peytchev 2007; Olson and Bilgen 2011). In particular, they are more likely to omit parts of questions, add words and phrases, give improper feedback, and not follow up with probes in response to uncertainty, yet they are also more likely to engage in rapport, develop better communication strategies, and adopt a more relaxed interviewing style, than less experienced researchers (ibid). Therefore, flexible interviewing continues to be practiced, in spite of the pressure to use standardised techniques. Skilled interviewing may therefore be more important than rigidly adhering to a particular technique. Belli et al. (2004) found that significant deviations from the survey script (i.e. those that alter question meaning) or not following up with a necessary probe was linked to poor data quality, whether standardised or flexible interviewing was the dominant technique. Hence, it is important to consider, not just the desirability of deviation from a standardised script, but also the helpfulness or otherwise of the content of those deviations.

## Building rapport in structured interviews

The ability to establish rapport is often considered to be one of the most important skills for effective interviewing. However, there is little consensus on how rapport should be defined in a survey context, or how rapport affects survey responses (Foucault et al. 2008). Capella (1990) describes rapport as a feeling of connection, mutual comfort, and conversational ease. Gremler and Gwinner (2000) describe rapport between interviewer and respondent in terms of ‘chemistry’ and being ‘in tune’. Rapport can be established through a range of interviewer behaviours, including highly attentive behaviour, common grounding behaviour (identifying mutual interests, finding similarities), courteous behaviour (honesty, civility, empathy), connecting behaviour (using humour, pleasant conversation, friendly interaction), and information sharing behaviour (giving advice, sharing knowledge, asking questions) (Gremler and Gwinner 2008). The extent of rapport has been measured in a variety of ways including asking interviewers and respondents whether they felt comfortable during the interview (e.g. Goudy and Potter 1975) and assessing respondent cooperation and interest Davis et al. (2010).

Whether rapport enhances data quality or not is also contested. For example, Weiss (1968) argues that rapport results in response bias because it causes respondents to ingratiate themselves to interviewers, encouraging distorted responses, especially on sensitive questions. In contrast, Holbrook et al. (2003) argue that rapport reduces response bias by motivating respondents to engage more deeply with the interview and give thoughtful, honest responses. Other researchers suggest that rapport has no systematic effect on data quality (e.g. Hensen et al. 1977), or that too little and too much rapport can be detrimental to data quality (Belli et al. 2001). However, appropriate sensitivity and adherence to everyday conversational norms is certainly necessary for any successful interview, and this is especially apposite in relation to household surveys conducted in respondents’ homes. Research by Lewis and Graham (2007) shows that, because being interviewed is an unfamiliar experience, respondents look to interviewers to help them feel ‘comfortable’. Being at ease is important for data quality, helping interviewees to answer honestly and openly (ibid) and to understand information and remember accurately (Ghosh et al. 2013).

Furthermore, there is also an important ethical dimension to this discussion. The subject matter of surveys can be sensitive and questions are often very personal, so it is very important to respond appropriately in order to avoid causing unintended harm to respondents. If a trauma is discussed with someone who is not empathic this can be psychologically harmful (see Kennedy-Moore and Watson 2001). Sensitivity and rapport also imply the need for flexibility. The capacity to respond flexibly is also ethically important when administering household surveys that may unearth sensitive information, since it is not possible to know in advance the kind of psychological associations that could be triggered by survey questions.

Hence, even in the administration of standardised survey instruments, researcher sensitivity to potential problems of mis-cognition or psychological distress is necessary, not least because such problems may not be explicitly voiced by respondents. Therefore, survey interviewers must be skilled in building rapport and using conversational techniques, even when expected to carry out standardised interviewing, so as to adequately and ethically respond to the unpredictable interview situation and to ensure that any deviations from the scripted interaction are productive in achieving valid and accurate responses. This implies that adequate training is imperative so as to equip the interviewers with sufficient skills in developing rapport and flexible interaction within the interview situation.

## Methodology

In the following analyses, we investigate the extent to which standardised interviewing protocols are, in fact, implemented and with what consequences for survey data quality and ethical research practice. The research drew on ‘paradata’, defined as ‘additional data that can be captured during the process of producing a survey statistic’ (Kreuter and Olson 2013, p. 3). Paradata include the bi-products of computer-assisted technology, audio recordings of the survey interview, and interviewers’ hand-written notes. There has been a rapid growth in the production and use of paradata over the last ten years (Durrant and Kreuter 2013), perhaps because researchers are now less likely to have direct contact with field interviewers, and therefore may be less likely to receive information about the conduct of fieldwork (Koch et al. 2009). Most survey data collection is contracted out and researchers are unable to influence the choice of interviewers or their training. Studying and analysing paradata is one way that we can find out more about the process.

In this paper we draw on interview transcript paradata collected as part of the *2012 Poverty and Social Exclusion in the United Kingdom main survey *(PSE-UK). The PSE-UK project included a household survey which re-interviewed respondents to the 2010/2011 Family Resources Survey. It was conducted on behalf of the University of Bristol (lead), Heriot-Watt University, The Open University, Queen’s University Belfast, University of Glasgow and the University of York by the National Centre for Social Research (NatCen) in Britain and the Northern Ireland Statistics and Research Agency (NISRA). The survey aims to improve the measurement of poverty, deprivation, social exclusion and living standards and, on this basis, to measure change in the nature and extent of poverty and social exclusion in the UK since 1999. Although only a single study is examined here, the field interviewers employed by the survey organisation for this study work on a variety of national household surveys, and we believe our findings are likely to be indicative of wider research practice in this area.

The PSE-UK study used a standardised data collection method. Although encouraging building rapport at the initial ’doorstep’ encounter, the PSE-UK survey organisation instructed interviewers to take a rigorously standardised approach during the interview. For example, interviewers were instructed to

- Ask the questions exactly as written—do not change any words...
- Read out the whole question right to the end, which is defined by the question mark...
- Never add anything to the question.
- Ask in the order on the questionnaire...
- Ask every question on your route—never miss out a question because you think it inappropriate.

(NatCen 2014, no page number)

After securing the consent of respondents and interviewers, a sample of 23 survey interviews were selected by the administering organisation for audio-recording. Since all the interviewers that agreed to participate were experienced, the sample was not randomly selected and this has been taken into account in the analysis. After audio-recording the process of administering these 23 surveys, the recordings were transcribed and anonymised. We then examined the extent and content of deviations from the scripted interview though (quantitative) behaviour coding of interviewer behaviour and (qualitative) framework analysis as outlined below.

### Behaviour coding

As well as helping to determine the effectiveness of the survey instrument, behaviour coding can also be used to enable researchers to learn more about how interviewers are performing the task. Behaviour coding approaches involve the application of a systematic coding framework to survey interview data (principally interview transcripts). The procedure examines interviewer/respondent interactions and within this research paradigm ‘problem free’ interactions are assumed to be those where interviewers read survey questions verbatim and respondents provided a code-able answer. As Willis (2005) notes, concerns have been raised about the subjectivity of the behaviour coding process. It is possible to offset this, to some extent, by using multiple coders and assessing the degree of inter-rater consistency or by taking extensive notes of decisions made. However, it must still be acknowledged that some degree of subjectivity is inherent to the behaviour coding process.

The behaviour codes used here were developed by amalgamating and adapting the coding frames used in previous behaviour-coding studies (e.g. Blair et al. 2007; Sala et al. 2008; Jurgenson and Childs 2011). The 23 interview transcripts were coded by a single researcher using the note-taking method. Table 1 shows the behaviour coding that was applied to the interviewer’s behaviour as recorded in the interview transcipts.

**Table 1:** Behaviour code

Interviewer		
Code		Description
EW	Exact wording	Interviewer read question exactly as worded
SC	Slight change	Interviewer read question with slight change that did not affect question meaning
MC	Major change	Interviewer made changes to the question that either altered, or could have altered, the meaning of the question
SQ	Skipped question	Interviewer entirely omitted an applicable question
NDP	Non-directive probe	Follow-up question/information was not leading (i.e. not suggesting a particular way of answering)
DP	Directive probe	Follow-up question/information was leading (i.e. suggesting a particular way of answering)
INI	Inaudible interviewer	Interviewer was not audible on the recording
AC	Adequate clarification	Explanation was in line with accurate interpretation
IC	Inadequate clarification	Explanation was not in line with accurate interpretation
IRI	Interprets response inadequately	Interviewer mishears, misunderstands etc.

### Framework analysis

Framework analysis (Ritchie and Lewis 2003) was used to conduct textual analysis of the interview transcripts implemented using proprietary NVivo Framework software. The distinctive aspect of framework analysis is that it allows themes to develop from the research questions, the reviewed literature, and the narratives of research participants. The process involved a number of distinct, interconnected stages: familiarisation with the data, identifying themes, indexing, charting and interpreting. The first stage, familiarisation with the data, was facilitated by behaviour coding all the transcripts initially. The second stage involved identifying themes in the data, using a hybrid of thematic analysis approaches, incorporating the inductive approach of Boyatzis (1998) and the deductive technique outlined by Crabtree and Miller (1999). Boyatzis’ approach involves a preliminary coding process to organize the data, and themes are then developed from these codes. Crabtree and Miller’s technique involves identifying codes in advance, based on existing theory and evidence, as well as a preliminary scanning of the text. The third and fourth stages, indexing and charting were facilitated by the NVivo programme. The final stage, interpreting the data, involved using (Krueger and Casey 2000) seven established criteria for interpreting coded data, considering: meaning; context; internal consistency; frequency and extensiveness of comments; specificity of comments; intensity of comments; and how data relate to the bigger picture.

## Results

### Behaviour coding

Table 2, below, shows the results of behaviour coding interviewers’ behaviours. The results demonstrate the variability in how strictly interviewers adhered to standardised interviewing protocols, with some closely following the scripted behaviour and other interviewers regularly deviating from the script and engaging in numerous digressions. To summarise, of the 1,635 questions asked, 55 % were read with the exact wording (EW), 37 % were read with a slight change (SC), 7 % were read with a major change (MC), and 1.6 % were skipped (SQ). When a probe was used, in 52 % of cases it was a non-directive probe (NDP), as considered to be ideal, but 48 % of the time a directive probe was used (DP). When a clarification was made in response to a request, 79 % of the time it was adequate and 21 % of the time it was inadequate (IC). On 1.8 % of occasions, the response to the question was inadequately interpreted (IRI).

**Table 2:** Results from behaviour coding the PSE survey process$^\mathrm{{a}}$

	EW	SC	MC	SQ	NDP	DP	INI	AC	IC	IRI
Int.1	48	28	3	2	0	0	0	1	1	0
Int.2	24	42	13	5	5	1	0	5	2	0
Int.3	16	38	8	1	0	1	0	8	0	1
Int.4	44	22	5	0	4	5	0	4	1	0
Int.5	24	32	4	1	0	4	1	2	0	0
Int.6	24	4	2	2	0	1	0	0	0	24
Int.7	58	13	1	0	3	6	0	3	3	1
Int.8	31	39	4	0	2	0	1	3	1	0
Int.9	47	21	0	1	5	1	1	3	0	0
Int.10	46	17	3	1	6	2	0	3	0	0
Int.11	62	16	1	0	0	2	0	3	0	0
Int.12	35	19	9	0	2	2	0	4	1	0
Int.13	40	22	14	1	6	1	0	3	0	1
Int.14	32	22	10	4	0	3	0	0	0	0
Int.15	36	40	8	1	0	1	0	4	1	0
Int.16	39	32	4	4	1	0	0	5	0	0
Int.17	57	25	2	0	1	1	0	3	0	0
Int.18	48	28	3	2	0	0	0	1	1	0
Int.19	50	16	0	0	2	4	0	3	1	0
Int.20	41	31	2	0	0	1	0	1	3	0
Int.21	26	34	11	0	1	0	0	1	1	1
Int.22	29	36	5	1	2	2	0	4	1	1
Int.23	34	27	2	0	3	1	0	2	1	0
Totals	891	604	114	26	43	39	3	66	18	29

$^\mathrm{{a}}$ The number of questions asked in each interview was different because not all questions applied to each respondent

Several authors propose that in standardised interviewing non-ideal interviewer behaviour (such as major changes to question wording, or skipping a question) should occur no more than 15 % of the time (Oksenberg et al. 1991; Fowler 1992). In this analysis, non-ideal interviewer behaviour (i.e. major change to a question, or skipping a question) occurred 9.9 % of the time (see Table 3). The range was between 0 % (interview 19) and 27 % (interview 2), indicating a wide variation of behaviours between the interviewer, though most fell below the 15 % threshold. A slight change to the question was classified with exact wording because the kinds of changes were minimal and did not affect meaning at all (for example, saying ‘something in a shop’ instead of ‘an item in a shop’). We focus here on interviewer behaviour only to ascertain the extent of deviation from a script and in combination with framework analysis we examine the nature of those deviations.

**Table 3:** Percentage of deviation (major change or skipped question) from standardisation (exact wording or slight change)

	EW	SC	EW+SC	MC	SQ	MC+SQ	%
Int.1	48	28	76	3	2	5	7
Int.2	24	42	66	13	5	18	27
Int.3	16	38	54	8	1	9	16
Int.4	44	22	66	5	0	5	7
Int.5	24	32	56	4	1	5	9
Int.6	24	4	28	2	2	4	14
Int.7	58	13	71	1	0	1	1
Int.8	31	39	70	4	0	4	6
Int.9	47	21	68	0	1	1	1
Int.10	46	17	63	3	1	4	6
Int.11	62	16	78	1	0	1	1
Int.12	35	19	54	9	0	9	17
Int.13	40	22	62	14	1	15	25
Int.14	32	22	54	10	4	14	26
Int.15	36	40	76	8	1	9	12
Int.16	39	32	71	4	4	8	11
Int.17	57	25	82	2	0	2	2
Int.18	48	28	76	3	2	5	6
Int.19	50	16	66	0	0	0	0
Int.20	41	31	72	2	0	2	3
Int.21	26	34	60	11	0	11	18
Int.22	29	36	65	5	1	6	9
Int.23	34	27	61	2	0	2	3
Totals	891	604	1495	114	26	140	9.9

Though there was a range of interviewer behaviours, the behaviour coding results suggest that in general the interviewers tended to make frequent deviations from the scripted questions even though they were given explicit instructions to use standardised methods. This level of flexibility is considered to be acceptable according to the literature (Oksenberg et al. 1991; Fowler 1992), with the overall average percentage of deviation from the script (not including minor changes) falling below the 15 % threshold.

### Framework analysis

With an overview of the *extent* of conversational diversion available to us from the behaviour coding, we then used a framework analysis of the transcripts to illuminate the *content* of these conversational diversions. Our interpretation reflects observable behaviour only since the motives for conversational behaviour cannot be readily ascertained. However, it is evident from this analysis that interviewers engaged in a range of conversational behaviours that were not directly relevant to answering the questions, including: giving emotional support; giving advice or information; complimenting the participant; making jokes; sharing the research process; and revealing personal information. We briefly describe and illustrate these below before reflecting on their wider significance in the concluding section.

#### Giving emotional support

In the 23 sample interviews, the interviewer was often appropriately responsive to the emotional material that arose and, at times, gave significant support to the participant. For example, in this excerpt, the interviewer gave the respondent plenty of space to talk and validated their feelings about some distressing news:

I: Your car?
F: Erm, I’m changing it, I’m supposed to be changing it today.
I: Are you?
F: Yes.
I: How exciting.
F: It’s tomorrow.
I: Oh. Have they phoned this morning and said it wouldn’t be ready?
F: No, my sister got diagnosed with lung cancer on Monday evening and she’s having a Petra scan later today, so I’m going to take her instead of picking my car up.
I: Oh well, you’d have to really wouldn’t you?
I: Is that, is it serious?
F: Yeah it’s lung cancer, so.
I: That is quite, still one of the...
F: Yeah. We’re just trying to find out where it’s spread to, so we’re at that stage.
I: Oh, I’m so sorry.
F: Yeah.
I: Where does she live, local, or?
F: [Name of town].
I: Oh right, not too...
F: Not too far.
I: Where’s she going?
F: [Name of hospital].
I: Oh right, that’s a good...
F: Yeah, yeah.
I: Good hospital there.
F: Yeah, she’s normally under [name of hospital] but that’s where she had the X-ray and the CT and now she’s been passed on.
I: Okay. That’s quite a worrying time isn’t it, ‘til you find out a proper diagnosis really.
F: Yeah and we can start...

The interviewer showed empathy and interest but still kept the situation reasonably ‘light’ by asking practical questions, rather than delving excessively into the emotions. The interviewer was quite prone to diversions, in general (24 % overall) so it could be that this was purely an example of her/his more chatty approach. However, in another example, interview 23, whilst the interviewer was much less likely to deviate from the script (just 3.28 % of the time), s/he still showed both flexibility and sensitivity. For example, in this excerpt s/he said:

I: Overall how satisfied [dog barks] are you [dog barks] with your life nowadays?
F: [dog barks loudly in background] Not satisfied [whispers].
I: No?
F: No [dog barks].
I: Oh, [name of respondent] [respondent becomes emotional] ...You miss your husband?
F: Oh [sniffs].
I: Was it some...
F: I wish I’d [never?] been married [unclear] and then I wouldn’t miss, I wouldn’t miss him [becomes emotional].
I: Oh [respondent sniffs] but then you’ve got your happy memories, haven’t you?
F: [whispers something inaudible] [sniffs]. Memories can be very painful [sniffs] yeah.
I: How long ago was it?
F: It’s over four years and it feels like forty sometimes and like yesterday other times.
I: Oh.
F: You know, 40, you think, since you last spoke to him in that sense and yet it’s as raw as if it was yesterday.
I: Sudden, was it sudden?...

Though this interviewer rarely deviated from the scripted interaction, s/he was able to be flexible when required. However, interviewers did not always display appropriate sensitivity or flexibility. For example, in the following exchange the interviewer moves on when a traumatic event is referred to and does not appear to respond at all:

F: I think I told you last time that my niece was murdered...
I: Oh right.
F: She was murdered three years ago.
I: Right.
F: DATE her killer was sentenced to 50 years and his sister
I: Yes.
F: SAME DATE gave birth to a little girl.
I: Okay.
M: I’ve got to the next question about household problems [unclear phrase] enter one to continue, so...
I: Yes, oh sorry, just press one.

Perhaps this interviewer demonstrated empathy non-verbally through tone of voice and expression. Relying on written transcripts meant that this non-verbal behaviour was not available to us. We also do not know the overall context, as clearly the interviewee had spoken about this during a previous interview (i.e. the Family Resources Survey interview). However, these words alone suggest little, if any, rapport, sensitivity or flexibility in this encounter. This interviewer did show a remarkably low level of deviation from the script overall (2 %) which, perhaps, indicates that their main concern was to carry out the standardised instructions they had been given. Bearing in mind that a lack of empathy in response to the disclosure of a traumatic event can be harmful (Kennedy-Moore and Watson 2001), this interview cannot be considered to be adequate or ethical. If this interviewee were re-traumatised by these comments, they may well be unlikely to feel motivated to disclose any other information that could leave them feeling vulnerable. Moreover, this example also illustrates that the way surveys are administered does not just pertain to data quality; it is also an issue of ethical conduct and common human decency.

#### Giving advice or information

This area of conversation included giving advice about financial matters, making proposals about how to resolve disagreements with neighbours, passing on ideas for entertaining children and giving advice about how to lobby the council for better services. For example, in the following interview advice is given regarding a financial matter relating to household insurance:

I: Oh, they’re nice clocks as well, aren’t they? Nice ones. Have you had them valued?
M: No. No I haven’t had them valued.
I: It might be an idea.
M: Yeah.
I: It’ll invalidate your contents insurance.
M: Oh right, okay.
I: I was told that the other day, if you’ve got something that’s not, that you haven’t mentioned, not only do they not pay out but they don’t pay out at all.
M: Oh right. Oh okay.
I: I haven’t checked that out but it came from quite a reliable source. You know somebody in sort of financial services. He said it’s wrong.
M: There’s a loophole for everything isn’t there?
I: Yeah, so...
M: I’ll look in to that.

Commonly, where both interviewer and respondent were parents, information and advice was given regarding caring for children. In this sample, the interviewer gave information and/or advice on school issues, relating to children at different life stages, hobbies, and affordable places to go with children. Here, for example, the interviewer discusses ideas for economically entertaining children:

I: Fantastic. Do you go to Star City? To the kid’s cinema?
M: No.
F: No.
I: £1.75 including the adults.
M: Oh right, blimey, yeah.
I: and I mean it’s ‘Chipwrecked’ at the moment, and we went, we paid full price to see it and it was like £24. We could have gone for, you can get the VIP, we go in the VIP seats and for three of us it’s £8.85 or something like that. So that’s on every Saturday and Sunday at 10:30.
F: Oh right.
M: Yeah.
I: That’s a really good, cheap...

Hence, here the interviewer engaged in several of the commonly recognised ways of building rapport i.e. common grounding behaviour (identifying mutual interests), connecting behaviour (pleasant conversation, friendly interaction), and information sharing behaviour (giving advice, sharing knowledge) (Gremler and Gwinner 2008). Whether building rapport was the interviewer’s conscious motivation or not, we might assume that this diversion would have such an impact.

#### Complimenting the participant

This kind of conversation involved recognising and praising the participant in relation to their home, their children, and their skills. For example, in interview 6, the interviewer said:

I: Yes [long pause, during which shuffling of papers can be heard. Female respondent returns to the room, door heard closing]. Did you make the papier-mâché pieces?
F: I did [chuckles].
I: They’re beautiful.
F: Do, do you like them?
I: Yes [laughs].
F: Yeah?
I: I think they’re lovely.
F: They started life as little pop, little pop bottles.
I: Sweet.
F: You know the little...
I: Yes.
F: Wee pop bottles you, children have? You put rice in it and then just pack it round with newspaper and masking tape and build a shape up. And then I, it’s, it’s actually mod roc, which is like plaster of Paris impregnated. Well, like a bandage really and you cut it and soak it and it behaves like papier-mâché.
I: It’s absolutely lovely.
F: Thank you [chuckles]. They were a bit fun.
I: I think they’re beautiful.

Here, again, the interviewer engages in behaviour which tends to encourage rapport, such as very attentive and connecting behaviours (friendly interaction) (Gremler and Gwinner 2008). If rapport enhances data quality, then we could assume this diversion from the script to have helped the data collection process. This is not to imply that the interviewer was being manipulative but, rather, engaging in the natural bonding behaviour that occurs when humans interact.

#### Making jokes

Jokes and humour occurred throughout many of the 23 sampled interviews. The jokes were initiated by the interviewer or the respondent and continued in humorous ‘banter’. This excerpt from one conversation is typical:

I: Okay, I’d like to ask some questions about your use of time. Card K1.
F: Has my husband sent you [laughing].
I: This is what the recordings for! [Laughing]
F: He’s been dying to find this information out for years [laughing].

[Both laughing]...

I: For the next question I would like you to imagine that you’ve come across an item in a shop, or on the internet, that you’d really like to have for yourself or to share with others in the household. It’s got a price tag of £150. It’s not an essential item for accommodation, food, clothing or other necessities, it’s an extra. If this happened in the next month how restricted would you feel about buying it?
F: I wouldn’t, one.
I: Number one.
F: Hence my Tefal ActiFry in the corner. [Laughing]
I: Have you used it?
I: Once or twice.
I: Once or twice, what the day you bought it and the day after that?
F: Hmm, probably [laughs].
I: It’s always the same isn’t it. Like a bread maker, oh yes.
F: Under the stairs.

[Both laughing]

Humour is a common feature of building and sustaining rapport (Gremler and Gwinner 2008). It is also well known for reducing stress (Martin 2007) which most would consider helpful to data collection because stress undermines the cognition and recall (Ghosh et al. 2013). Therefore, we could assume that stress relieving laughter would increase data quality as both interviewer and interviewee will be more able to think clearly, despite discussing sensitive topics.

#### Sharing the research process

Another category of conversation could be described as ‘sharing the research process’. This included expressing frustrations about the work; discussing the process of carrying out the survey, including mistakes made in the past; talking about findings from the survey; and providing some background about the survey development. For example, in relation to expressing frustration about the work, one interviewer (who infrequently answered the question as worded) answered a question from the respondent in the following way:

F: How many people will you be seeing tonight?
I: Oh, well if that computer’s giving me a bit of jip, I’ll... I’ll call it a draw. I still say there’s something wrong with it and they keep saying, oh it’s just the security. Every computer I’ve, the one before that was brilliant. It used to start up in no time and...
F: Yeah.
I: ...Never had any trouble with it. This one keeps freezing.
F: Not fun is it?
I: No, no. I mean, if it was my own I might just throw it from somebody’s, somebody’s window.

In relation to talking about the study findings, this appeared to be undertaken in order to try to help the respondent think about or understand the question. However, sometimes the comments made could be considered to be leading the respondent. For example, in the following interaction, an interviewer said:

I: By approximately how much has your income decreased since you were last interviewed?
F: That’s a really difficult one to answer.
I: Give a best estimate really. What they’re trying to do is get, I mean most people are saying their housing costs have increased, literally all the ones I’ve interviewed, and then most people have either stayed the same or decreased income wise. So obviously that’s a double, double effect then.

A particular aspect of conversation that could become undermine data quality occurred where the interviewers referred to other interviews they had undertaken. NatCen instructs their interviewers in ethical research practice, stating that interviewers should respect confidentiality and take care that in casual conversations they do not repeat ‘interesting’ tales about respondents (NatCen 2014). In spite of this, on this occasion, the interviewer made the respondent they were talking about identifiable:

I: I interviewed a family of four yesterday and they weren’t on, in a great area, what do they call, like a council house. Two children living at home but they were all working and no benefits at all. Which is really unusual, not even child benefit because the children were too old. And, erm...
F: It’s not that unusual, because when we were growing up that’s what happened to us.
I: It’s very unusual now.
F: [laughs] Is it?
I: I interview 40 or 50 households a month and it’s very unusual, very, very unusual. And, erm, but I mean one was, the daughter was a team leader of 30 odd in this pick and pack place for [NAME OF WORKPLACE], and had to work bank holidays as unpaid, one shift every bank holiday is unpaid, and take home £1400/£1500 a month. It’s a lot of work for...

Sometimes the interviewer even referred somewhat derogatively to other interviewees. For example, on this occasion, one interviewer said:

I: Excellent, that was very quick...This is my last one tonight, if I’d have had that man as my last one...
F: You’d still be doing it.
I: You can’t really, if they want to talk, you know. He’s got a few problems and if he wants to talk then you, sort of, feel half obliged to listen.
F: Oh yes, especially when they’re on their own.
I: But this is great for a last one, somebody decisive.

Therefore, on these occasions sharing the research process may have undermined survey data quality. It may have provoked mistrust on the part of the interviewee as they wondered what might be repeated or said about themselves. Again, though, it is not just a question of data quality, there is an ethical dimension. Though the flexibility to digress from rigid standardisation seems important, as earlier examples illustrate, this has to be done skilfully in order to ensure the greatest degree of accuracy of the survey and to avoid causing harm to research participants.

## Revealing personal information

Some commentators argue that interviewers should share information about themselves with participants as this helps to equalise the researcher-participant relationship and create rapport (e.g. Johnson 2002). Others have found that, research participants, while finding it helpful if the interviewer’s personality comes across, do not want the interviewer to share personal information with them (Lewis and Graham 2007). In the latter study, respondents to household surveys felt that knowing something personal about the interviewer might influence the answers they gave; and increase their desire to withhold information. NatCen’s interviewers training manual explicitly states that the interviewer should take care when asked questions about themselves because their answers could bias the results. They are encouraged to give a non-committal reply when this happens and encourage the respondent to talk about themselves again (NatCen 2014)

Nevertheless, personal information was often revealed by the interviewers that we audio-recorded. Although the reasons were not always obvious, in general it seemed to be an attempt to put the respondent at ease, perhaps as a subconscious interviewer strategy to help to promote some balance in the interaction in terms of the extent of personal self-disclosure. Some of the revelations included anecdotes about personal failures, perhaps to encourage the respondent to feel less intimidated or more willing to ‘open-up’. At times, revealing personal information seemed to be an attempt to establish some common ground. Though this is considered an effective way to build rapport, sometimes this strategy can at times appear somewhat desperate, thereby undermining rapport, as in the following example:

I: How old is this one?
M: She’s, she’ll be six in July.
I: Oh, yes, they do that. Which date in July, do you remember?
M: Pardon?
I: What’s her, what’s her birthday? Just I’m a July birthday.
M: I think it’s 9th of July. I’ll have to look on the calendar.
I: That’s my, my friend from university is on the 9th and I’m on the 21st.

Sometimes, the dialogue seemed to be a form of ‘nervous chatting’ or ‘thinking aloud’, since some of the discussion elicited no obvious interest from the respondent. For example, in the following extract, without being asked any question on the topic, the interviewer spontaneously began to talk about his cars. Whether these personally revealing interactions helped or hindered the interview process is hard to establish but, at face value, they do not seem to help in establishing and maintaining rapport, as they seemed to be out of synch with the needs of the respondent:

I: I drive; I do a lot of mileage. So I drive old cars now because I’ve wrecked two or three good cars [male respondent laughs]. I’ve got the estate car which I normally use when I’ve got my dogs. And I use the little car when I’m doing longer distances ’cause it’s cheaper to run.
M: Yeah.
I: But they’re old cars and I’ll drive them ’til they’re finished
M: Yeah.
I: and get the scrap value, get another one
M: Yeah.
I: and then when I’m not doing as much mileage, I’ll probably get a better car unless I win the lottery.
M: Yeah.

Despite being given strict instructions to take a standardised approach, these experienced interviewers introduced a great deal of unscripted conversation and often deviated from the exact question wording. The framework analysis themes correspond closely to the themes identified in the literature on ‘rapport building’, i.e. very attentive behaviour, common grounding behaviour, courteous, connecting behaviour, and information sharing behaviour (e.g. Gremler and Gwinner 2008). Hence, we could assume that the interviewers were attempting to build rapport, or perhaps just ‘being themselves’ so as not to introduce too much artificiality, with accompanying tension, to the process. Sometimes, this may have helped the interview to flow more naturally, but occasionally the interviewer seemed to behave in ways that were potentially harmful, both to the research and to the participant.

## Discussion, conclusion and implications

Various degrees of purely conversational or standardised interviewing techniques are available to survey researchers. Both techniques may improve response accuracy in some circumstances, but there are always associated costs. Conversational interviewing clearly increases the duration of interview. It can also mean that training the interviewer can take longer so as to ensure that they really understand the nature of the survey and the concepts being used. Yet, with standardised interviewing, questions may be misinterpreted, and it is more difficult to answer accurately where there are atypical situations. As outlined earlier, some suggest that conversational interviewing, with its emphasis on rapport, motivates respondents to answer more openly and honestly; and others argue that it encourages respondents to ingratiate themselves with interviewers, and choose answers that will avoid embarrassment.

Although, this analysis is not informative regarding the impact of interviewing technique on survey response, it does illustrate the wide variety of interviewing approaches adopted in the field, even when interviewers are instructed to use standardised interviewing. Whether conversational or standardised approaches are adopted, interviewer skill is clearly vital in promoting response accuracy, in facilitating respondents’ cooperation, and in safeguarding the ethical conduct of survey research. Whilst insensitive behaviour by interviewers may be rare it is somewhat concerning that this is evident in such a small sample, and among interviewers who had consented to being recorded. This emphasises the need for further monitoring and evaluation of the content of standardised interview conversations and the importance of training in conversational techniques for field interviewers even when delivering standardised questionnaires.

However, several potentially confounding factors need to be noted with regard to interpreting the data. Firstly, we do not know whether the approach used when the interview was audio-recorded was the interviewer’s usual style or their reaction to the specific respondent. We did not collect data on the range of behaviour of the individual interviewers, which would provide information on this. Secondly, although interviewers were non-randomly selected and experienced, the potential biases this may introduce are unknown. Thirdly, the interviews were recorded, potentially introducing a number of related ‘interviewer effects’ into the process. The interviewers may have been more motivated to read the questions exactly as worded and to behave more consistently with the expectations of researchers to be neutral, friendly and professional than they normally would be, since they were conscious of being monitored. Given that we were observing the practices of experienced interviewers who were aware of being monitored, we might expect more adherence to the instructions to carry out standardised interviewing than is generally the case. Yet interviewer digression was extensive and not always skilled. Therefore, it is possible that the extent of sometimes inappropriate interviewer deviation from the scripted interaction is, in fact, even more common than suggested here.

Having identified the extent and content of interviewer digression, we do not advocate a more standardised approach. All of the interviewers sampled, at some point, took a conversational approach and this was often carried out in a skilled manner. Some of their comments were leading and insensitive, possibly impacting on survey reliability. Furthermore, on occasion, their comments appeared to be insensitive to the point of possibly being harmful to the respondent. The survey organisation’s training appeared to focus primarily on developing interviewer’s standardised interviewing skills as a way of averting potential problems relating to reliability and ethicality. This may have left interviewers insufficiently prepared with regard to situations where more conversational interviewing skills were needed. The requisite flexibility which would have helped to enhance reliability and ethicality in those moments was, therefore, occasionally lacking. Whilst it could be argued that the problem is more in relation to the interviewers not following the instructions they were given, than the training itself, we would assert that the training should be sufficiently thorough that the interviewers are aware of the importance and meaning of ‘confidentiality’ and ‘rapport’ etc. We might assume that they thought their interview technique was adequate since they were, on this occasion, being monitored, and so their lapses were a result of a lack of information, rather than a lack of will. The interviewers seemed to be attempting to use standardised procedures that were inadequate for the some of the situations that they faced.

Because of the aforementioned emphasis on developing the ideal survey instrument, it is understandable that survey administration organisations should focus on training their interviewers to conduct standardised interviews. Yet, the analysis carried out here underlines the importance of building appropriate rapport in interviewer–respondent interactions in a survey setting. Therefore, survey administration organisations may want to consider the extent to which existing interviewer training provides sufficient weight to the development of the kinds of conversational interviewing techniques necessary to developing and maintaining rapport. They may also wish to consider retraining more experienced interviewers, as these may be more likely to make inappropriate comments (e.g. Pickery and Loosveldt 2001; Olson and Peytchev 2007).

This paper highlights the complex rapport and sensitivity skills that field researchers require, even when administering standardised surveys. It, therefore, contradicts a trend towards eliminating ‘interviewer effects’ through ‘virtual human interviewing’. Foucault et al. (2008), for example, argue that preliminary evidence suggests that virtual human interviewers increase the likelihood that respondents will agree to participate in and complete web-based surveys, and give honest, accurate responses to even sensitive survey questions. These authors suggest that if virtual human survey interviewers are to be truly effective, we need to better understand how virtual humans should interact with real human respondents. Such a proposal ignores the apparent need for flexibility in interviews that we have highlighted in this paper. However, what may be rapport for one respondent could be perceived as ‘over-friendliness’ by another; what may be aloof behaviour for some, could be interpreted as professional neutrality by others. Lewis and Graham (2007) found that to avoid a negative interview experience, research participants emphasised the importance of the interviewers’ ability to respond effectively to the specific individual and particular situation. The research participants particularly found it hard to give advice about how the interviewer should respond when someone became upset as they felt this required the utmost sensitivity to the particular situation. Survey interviewers are required to be attentive to what is said, the context, and even the largely non-verbal, even subconscious messages are being transmitted by the respondent. All this requires a greater degree of sensitivity, skill and flexibility than virtual humans can achieve, according to the ‘Turing test’ (Turing 1950). The analysis outlined in this paper shows that this degree of skill and sensitivity is sometimes difficult even for real humans to achieve. Therefore, as an additional observation, we do not think virtual human interviewing is the way forward for increased accuracy, cooperation and ethicality in household surveys.

Another implication of this study relates to the perceived division between quantitative and qualitative methodologies. Despite continuing attempts to see the two as paradigmatically different and based on incommensurate ontological, epistemological and methodological assumptions (e.g. Sale et al. 2002), such divisions are increasingly seen as a false dichotomy. We have provided an example of how quantitative data collection has qualitative elements (i.e. the content of unscripted conversations) and how these qualitative elements can be quantified (i.e. the numbers of conversational digressions). In current research practice, this traditional qualitative/quantitative division is breaking down rapidly, facilitated by technological innovation. Many computer-assisted qualitative data analysis software programs now, not only enable qualitative coding to be carried out, but also allow the material to be exported to quantitative software programs for subsequent statistical analysis. At the same time, statistical software developers are now developing text analysis programmes. Some researchers now advocate that we teach research methodology courses without dividing quantitative and qualitative methods and they even propose removing the ‘Q words’ from text-books (e.g. Onwuegbuzie and Leech 2005). In this vein, Greene and Hall argue:

To use the qualitative and quantitative labels for paradigms is to reify and essentialize them and thereby disregard their constructed nature and discount the diverse histories and social locations of different kinds of qualitative and quantitative inquiry (2010, pp. 124–125)

Recognising the value of using both quantitative and qualitative methods, numerous studies now employ both techniques. The PSE-UK project itself does so. However, it is evident that there are qualitative aspects of the quantitative and quantitative aspects of the qualitative even within mixed-methods studies. Quantitative research, when viewed as a distinct paradigm from qualitative research, is often linked to the assumption that it is independent of the inquirer. It is generally considered that the data is (or should be) undistorted by contexts that are not included as variables. This paper illustrates how quantitative research struggles to achieve this and, therefore, is closer to qualitative research than is generally recognised. Perhaps, it is ultimately time to fully acknowledge the existence and value of qualitative computations and quantitative conversations.
